# Prognostic Value of Post-Cerclage Transvaginal Ultrasound Parameters in Predicting Spontaneous Preterm Birth

**DOI:** 10.3390/medicina61122111

**Published:** 2025-11-27

**Authors:** Gul Alkan Bulbul, Emine Kirtis, Hulya Kandemir, Busra Tsakir, Cem Yasar Sanhal, Ibrahim Inanc Mendilcioglu

**Affiliations:** 1 Department of Gynecology and Obstetrics, Division of Perinatology, Faculty of Medicine, Akdeniz University, Antalya 07070, Türkiye; edogru07@hotmail.com (E.K.); dr_hulyaeren@hotmail.com (H.K.); cemsanhal@gmail.com (C.Y.S.); imendilcioglu@gmail.com (I.I.M.); 2 Department of Perinatology, Antalya Training and Research Hospital, Antalya 07070, Türkiye; busra_dgn@hotmail.com

**Keywords:** cerclage, cervical length, funneling, intra-amniotic sludge, preterm birth, transvaginal ultrasound

## Abstract

*Background and Objectives:* Preterm birth (PTB) remains a leading cause of neonatal morbidity and mortality worldwide, particularly among women with cervical insufficiency. This study aimed to identify whether transvaginal sonographic parameters assessed following McDonald cerclage could act as predictors for the risk of spontaneous PTB < 34 weeks. *Materials and Methods:* A cohort of singleton pregnancies without structural abnormalities that underwent McDonald cerclage and had at least one transvaginal ultrasound (TVUS) examination between 16–25 weeks’ gestation was retrospectively analyzed. Two blinded reviewers evaluated the images. Measurements included total cervical length, cervical lengths above and below the stitch, anterior and posterior cervical widths at the suture level, as well as anterior and posterior stitch depths. Additionally, the angle between the cervical canal and the anterior uterine wall was assessed at both the internal and external os. Presence of funneling and intra-amniotic sludge was also noted. Maternal demographic and obstetric data were collected, and ultrasound findings were compared between women who delivered before and after 34 weeks. *Results*: A total of 45 women were enrolled, with cerclage indications categorized as history-based (76%), ultrasound-based (9%) or exam-based (15%). Overall, PTB < 34 weeks occurred in 38% (*n* = 17). Maternal characteristics did not vary between groups. However, both total cervical length and cervical length above the stitch were significantly shorter in women with PTB < 34 weeks vs. PTB ≥ 34 (27.60 ± 8.81 mm vs. 35.89 ± 7.09 mm, *p* = 0.012; and 13.15 ± 9.17 mm vs. 21.87 ± 8.95 mm, *p* = 0.004, respectively). Funneling beyond the cerclage was observed exclusively in women who delivered < 34 weeks (29.4%, *p* = 0.005). Funneling at the internal os (58.8% vs. 3.6%, *p* < 0.001) and intra-amniotic sludge (29.4% vs. 3.6%, *p* = 0.023) were likewise more frequent in this group. *Conclusions*: In addition to cervical length measurement, post-cerclage transvaginal ultrasound—through the evaluation of suture position, cervical funneling, and intra-amniotic sludge—may assist in identifying women at higher risk of PTB < 34 weeks.

## 1. Introduction

Cervical insufficiency is characterized by the premature inability of the cervix to maintain structural integrity before the onset of labor, predisposing women to preterm birth (PTB). The condition is reported in 0.2–7% of pregnancies and contributes to about 8% of recurrent pregnancy losses in the second and third trimesters [1]. Although the underlying pathophysiology remains incompletely understood, cervical cerclage is proposed to mitigate the risk of PTB in appropriately selected individuals by rein-forcing the cervix mechanically and preserving its functional integrity. This mechanical support may also prevent ascending infection by maintaining cervical length and the protective mucous plug [2]. Cerclage procedures are categorized based on clinical indication into history-indicated, ultrasound-indicated and exam-indicated. History-indicated cerclage is generally performed prophylactically in asymptomatic women, typically between the 14–16 weeks’ gestation, based on a history of one or more prior second-trimester losses attributed to presumed cervical insufficiency. Ultrasound-indicated cerclage is generally placed between the 16–24 weeks’ gestation in women with a prior spontaneous PTB and a transvaginal cervical length < 25 mm, while exam-indicated cerclage is placed when cervical dilation ≥ 1 cm is detected on examination [1,3]. Among the available surgical approaches, the McDonald and Shirodkar techniques are most widely utilized. Both involve suturing performed around the cervix to reinforce its closure and delay delivery, although they differ in technical nuances regarding suture placement and dissection [4].

Numerous transvaginal ultrasound (TVUS) parameters have been explored in attempts to predict PTB following cervical cerclage. These include measurements such as total cervical length, anterior and posterior cervical widths at the level of the suture, the distance between the cerclage suture and the internal and external os, the distance from the suture to the cervical canal and the uterocervical angles [5,6,7,8,9]. Despite these investigative efforts, the results across studies remain inconsistent, with considerable heterogeneity in findings [7,10,11,12,13]. Major limitation in the existing literature is the variability in study populations, particularly regarding gestational order, as well as discrepancies in the cerclage technique employed (i.e., McDonald vs. Shirodkar). Additionally, specific TVUS features—such as the presence of amniotic fluid sludge (AFS), characterized by echogenic particulate matter suspended within the amniotic cavity, and cervical funneling—have been linked to a heightened risk of PTB [14]. Nevertheless, the prognostic value of these findings in the context of cerclage remains uncertain due to the conflicting interpretations reported across studies [6,15]. Given these inconsistencies, the aim of this study was to evaluate the prognostic significance of TVUS parameters obtained after McDonald cerclage placement in identifying women at risk for spontaneous PTB < 34 weeks.

## 2. Methods

### 2.1. Study Population

This retrospective cohort study was performed at the Department of Perinatology, Akdeniz University Hospital—a tertiary care center in Antalya, Turkey—between October 2019 and May 2022. Women with singleton pregnancies who had undergone McDonald cerclage were identified using the hospital’s electronic medical record system. All eligible patients, regardless of the cerclage indication, were considered for inclusion. Cerclage indications were categorized as follows: history-indicated cerclage was defined as placement following one or more prior second-trimester losses attributed presumably to cervical insufficiency; ultrasound-indicated cerclage was performed among women with previous spontaneous PTB and a sonographically measured cervical length < 25 mm between 16–24 weeks’ gestation; and exam-indicated cerclage was performed in cases of painless cervical dilation ≥ 1 cm detected during the second trimester.

The participants were included if carrying a singleton fetus without structural anomalies, had received a McDonald cerclage, underwent post-cerclage transvaginal cervical length (TVCL) surveillance and delivered at the study institution. Exclusion criteria included multiple gestations, fetuses with genetic or major structural anomalies, absence of post-cerclage cervical ultrasound, or delivery outside the institution. The study was approved by the Akdeniz University Institutional Ethics Committee (Approval No: KAEK-325 dated 11 May 2022).

### 2.2. Cerclage Procedure and Follow-Up

In our institution, McDonald cerclage is preferred due to its simpler and less invasive nature, which allows easier placement, removal, and better sonographic visualization of the suture site during follow-up compared with the Shirodkar method [16]. Cerclage procedures are primarily performed by maternal–fetal medicine specialists, with the assistance of resident and fellow trainees. The procedure is performed in the operating room under epidural or general anesthesia, using a single 5 mm Mersilene tape suture (Ethicon, Inc., Somerville, NJ, USA) placed circumferentially around the cervix.

Cervical cerclages are generally removed at 36–37 weeks’ gestation unless earlier removal is indicated due to intraamniotic infection or spontaneous preterm labor. Following cerclage placement, patients routinely undergo TVCL surveillance beginning approximately one to two weeks postoperatively. Moreover, vaginal progesterone is administered at a dose of 200 mg daily based on standard obstetric criteria, particularly among women with previous spontaneous PTB or in nulliparous women with a cervical length < 20 mm. Notably, during the study period of this retrospective study, 17-hydroxyprogesterone caproate (17-OHPC) was still in clinical use and prescribed accordingly, as it had not yet been withdrawn from the market.

### 2.3. Ultrasound Measurements

**Figure 1:**
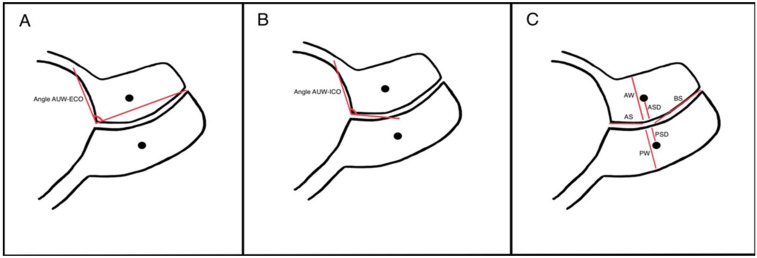
Representative illustration of measurements obtained from transvaginal ultrasound of the cervical canal and lower uterine segment in the sagittal view at the maternal midline. (**A**) Uterocervical angle between the anterior uterine wall and external cervical os (AUC-ECO). (**B**) Uterocervical angle between the anterior uterine wall and internal cervical os (AUC-ICO). (**C**) Cervical lengths above the stitch (AS) and below the stitch (BS), anterior stitch depth (ASD) and posterior stitch depth (PSD), anterior cervical width (AW) and posterior cervical width (PW) at the level of the stitch.

All ultrasound examinations were performed using the Voluson E8 system (GE Healthcare Austria GmbH & Co OG, Zipf, Austria), and images were analyzed using the integrated ultrasound software (Voluson E8 BT19–BT21). Stored post-cerclage TVUS images were reviewed retrospectively by two blinded reviewers. To ensure consistency and image quality, optimal scans were selected based on the Cervical Length Education and Review (CLEAR) guidelines and subsequently analyzed using accordingly [17]. In addition to the standard cervical length measurement, a comprehensive assessment of additional cervical parameters was performed using predefined measurement protocols. These parameters included cervical lengths above the stitch and below the stitch, as well as anterior and posterior stitch depths—defined as the linear distance from anterior stitch to the cervical canal and from the posterior stitch to the cervical canal. Cervical widths at the level of the stitch were measured from the anterior-most and posterior-most margins of the cervical stroma to the cervical canal. Furthermore, two distinct uterocervical angles were calculated: one be-tween the anterior uterine wall and a line extending from the internal to the external os, and another between the anterior uterine wall and a line corresponding to the axis of the proximal cervical canal [6]. Figure 1 provides a representative illustration of measurements obtained from TVUS of the cervical canal and lower uterine segment. Sonographic signs such as AFS, which is defined as echogenic, dense particulate matter located near the internal os and cervical funneling (characterized by protrusion of the amniotic membranes within the cervical canal and measured along its lateral edge) were also recorded when present [18].

Additional demographic and obstetric variables were obtained from electronic medical records including maternal age, pre-pregnancy body mass index (BMI), gravidity, parity, history and gestational age of the earliest prior spontaneous PTB (in multiparous women), as well as gestational age at cerclage placement, cerclage indication and use of progesterone therapy. The primary outcome was defined as spontaneous PTB < 34 weeks, which is considered early preterm birth and is strongly associated with cervical shortening and adverse neonatal outcomes [19,20].

### 2.4. Statistical Analysis

Demographic features, baseline clinical data, and sonographic parameters were analyzed by comparing two groups. Normality of continuous variables was assessed using the Kolmogorov–Smirnov test. Normally distributed data were expressed as mean ± standard deviation and analyzed by the independent samples *t*-test, whereas non-normally distributed data were presented as median (interquartile range) and examined with the Mann–Whitney U test. Furthermore, categorical variables were assessed with either the Chi-square test or Fisher’s exact test, where appropriate. In all analyses, a *p*-value < 0.05 was regarded as statistically significant, and statistical procedures were conducted using IBM SPSS Statistics for Windows, Version 25.0 (IBM Corp., Armonk, NY, USA). In the post hoc power analysis, the power of the study was found to be 81% with an effect size of 0.9 for cervical length measurement.

## 3. Results

**Figure 2:**
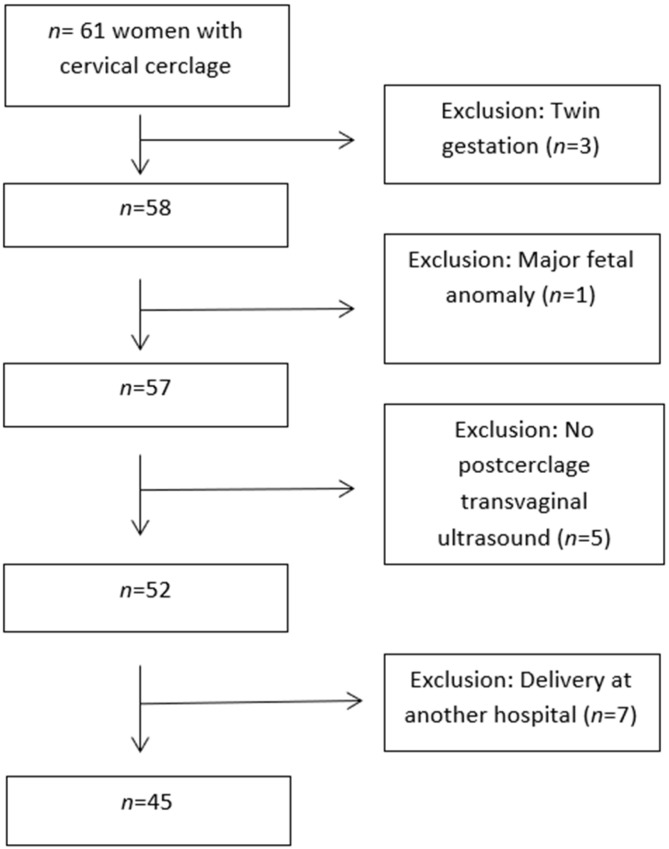
Flow diagram of study cohort.

During the study period, 61 pregnant women who had undergone McDonald cerclage were initially identified. Of these, three were excluded at an early stage—two due to twin gestations and one due to a major structural anomaly. An additional five participants were excluded due to the absence of post-cerclage ultrasound data, and seven were excluded because delivery occurred at an outside facility. After these exclusions, 45 women met the study criteria and were analyzed in the final cohort (Figure 2).

Among the study cohort, 34 women (76%) received a history-indicated cerclage, 4 (9%) underwent ultrasound-indicated cerclage and 7 (15%) received an exam-indicated cerclage. The incidence of PTB < 34 weeks was 38% (*n* = 17), with only a single case delivering before 24 weeks. The maternal demographic and clinical features of the cohort are summarized in Table 1. No significant association was found between PTB < 34 weeks and maternal age, smoking status, pre-pregnancy BMI, or gestational age at prior PTB among multiparous women. Similarly, the administration of vaginal progesterone or 17-OHPC did not significantly differ between those who delivered before and after 34 weeks.

Ultrasound findings were analyzed by comparing women who delivered < 34 weeks (*n* = 28) with those delivering ≥ 34 weeks (*n* = 17) (Table 2). The mean gestational age at TVUS examination did not differ significantly between the groups (19.6 ± 4.2 weeks vs. 18.6 ± 4.5 weeks, *p* = 0.629). The interval from cerclage placement to the first ultrasound was similar between the groups, with a median of 14 days in both (range: 7.0–35.0 vs. 3.0–28.0 days; *p* = 0.675). Total cervical length and cervical length above the stitch were significantly lower in women who delivered < 34 weeks compared to the women who delivered ≥ 34 weeks: (27.60 ± 8.81 mm vs. 35.89 ± 7.09 mm, *p* = 0.012) (13.15 ± 9.17 mm vs. 21.87 ± 8.95 mm, *p* = 0.004), respectively. However, no significant differences were observed in other TVUS parameters (such as the uterocervical angles, cervical length below the stitch, anterior and posterior cervical width at the level of the stitch, anterior and posterior stitch depth) between the two groups. Notably, funneling of the membranes to or beyond the level of the cerclage suture was detected in 29.4% of the women who delivered < 34 weeks, whereas it was absent in those who delivered ≥ 34 weeks (*p* = 0.005). Likewise, funneling at the internal os was significantly more prevalent in women who delivered < 34 weeks compared to those who delivered ≥ 34 weeks (58.8% vs. 3.6%, *p* < 0.001). Additionally, the presence of AFS was observed at a significantly higher rate among women who delivered < 34 weeks compared to the ones who delivered ≥ 34 weeks (29.4% vs. 3.6%, *p* = 0.023). Table 2 presents the TVUS parameters obtained at the first follow-up after cerclage, compared between women who delivered < 34 weeks and those at ≥34 weeks.

## 4. Discussion

Building upon previous research, the present study investigates the association be-tween early post-cerclage TVUS findings and the risk of PTB < 34 weeks in women who underwent McDonald cerclage. The results demonstrate that specific sonographic features observed during the first follow-up scan—including a short cervical length, short cervical length above the stitch, membrane funneling at or beyond the cerclage and at the level of the internal os, and AFS—were all significantly associated with an elevated risk of PTB < 34 weeks.

Cervical length screening using transvaginal ultrasound is generally recommended for singleton pregnancies with a history of spontaneous PTB, although it is not routinely performed after cerclage placement [21,22]. Nevertheless, consistent with our findings, recent studies have indicated positive recognition in post-cerclage ultrasound measurements of cervical length [5,6,8,9,11,13]. Evidence suggests that the risk of PTB increases progressively as the post-cerclage cervical length shortens. Therefore, prophylactic cerclage may reduce the risk of PTB by restoring cervical anatomy and reinforcing the cervical barrier function, thereby preventing ascending infections [9].

Although some earlier studies have suggested that placing the cerclage suture at a higher level above the external cervical os may be associated with prolonged gestation [7,12], other investigations have failed to confirm this relationship [5,11]. In our study, the cervical segment above the cerclage stitch was significantly shorter in women who experienced PTB <34 weeks compared to those who delivered ≥ 34 weeks. Our findings suggest that, particularly in women with a shortened cervix, the residual cervical length above the cerclage may play a crucial role in predicting the risk of PTB. Supporting this hypothesis, Bachar et al. proposed that pressure exerted by the amniotic sac and the fetus may result in an “inside-out” pattern of cervical dilation, initiating at the internal os and progressing downward toward the level of the cerclage, thereby leading to progressive cervical shortening [5].

Contrary to the findings of Battarbee et al., our study did not demonstrate significant differences in either the anterior or posterior cervical width at the level of the stitch, or in the distance between the suture and the cervical canal, when comparing women who delivered < 34 weeks with those who delivered ≥ 34 weeks [6]. In Battarbee’s study, it was hypothesized that placement of the suture too close to the cervical canal might fail to adequately support the surrounding cervical stroma and could potentially trigger an inflammatory response in the cervical mucosa, thereby increasing the risk of PTB. The difference between our findings and those presented by Battarbee et al. is likely to be explained by methodological differences specifically, our exclusive inclusion of McDonald cerclage procedures as well as potential ethnic or demographic variations in the study populations. The study by Battarbee et al. included 102 women from a multiethnic Western European population, whereas our cohort consisted of 45 Turkish women from a single tertiary center. The rate of preterm birth < 34 weeks was slightly higher in our study (37.8% vs. 27.5%), with comparable overall preterm birth rates < 37 weeks (48.9% vs. 47%). In addition, the mean maternal BMI was higher in the cohort reported by Battarbee et al. [6].

The cervix, composed mainly of dense collagen and supported by uterosacral and cardinal ligaments, plays a crucial role in maintaining uterine Integrity during pregnancy. TVUS enables assessment of cervical function through measurements such as cervical length and uterocervical angle. It has been proposed that wide uterocervical angle might allow more direct transmission of intrauterine pressure to the cervix, potentially promoting cervical shortening and dilation, whereas a narrower angle may shield the internal os from excessive mechanical stress [23,24]. While earlier studies have reported a potential association between a wider uterocervical angle and an increased risk of PTB [23,24]; this relationship was not confirmed in the post-cerclage setting [6]. Consistent with those findings, our study also demonstrated no significant association between post-cerclage uterocervical angle and the risk of PTB. We speculate that the cerclage procedure itself may alter the anatomical configuration of the cervix, thereby reducing the predictive value of uterocervical angle measurements after suture placement.

In our study, the presence of membrane funneling to or beyond the level of the stitch, as well as at the internal os, was notably more frequent in the early PTB group. Among post-cerclage ultrasound findings, cervical funneling is the most consistently reported parameter associated with PTB in women with cerclage [6,15,24]. Post-cerclage funneling to the stitch may indicate that elevated intrauterine pressure weakens the membranes at their most dependent position against a fixed cervix, potentially increasing the risk of PTB [25].

AFS is identified on TVUS as free-floating echogenic material located near the internal os. This finding is regarded as a marker of microbial invasion into the amniotic cavity, which has been correlated with higher rates of preterm prelabor rupture of membranes, PTB and neonatal infection [26]. In our cohort, AFS was significantly more prevalent among women who delivered before 34 weeks, consistent with the findings of Battarbee et al. who similarly reported a higher prevalence of sludge in women who de-livered preterm [6]. However, this association has not been consistently reported in the literature. A retrospective cohort study including 177 women undergoing McDonald cerclage found no significant relationship between the presence of AFS and PTB. In that study, although 60 patients exhibited sonographic evidence of sludge, no differences were observed in mean gestational age at delivery or in the rates of PTB at <28, <30, <32, or <36 weeks compared to those without AFS [27]. Similarly, Syeda et al. reported that the presence of AFS did not predict adverse neonatal outcomes and was not significantly associated with risk factors among patients who received exam- or ultrasound-indicated cerclage [28].

The impact of obesity on cerclage efficacy remains insufficiently explored. In a large retrospective analysis, Nguyen et al. reported that while maternal obesity did not increase the risk of spontaneous PTB, it was associated with a higher overall rate of medically indicated PTB, suggesting that cerclage remains effective across BMI categories [29]. In our study as well, no significant association was found between maternal BMI and PTB < 34 weeks. Similarly, consistent with previous research, our study also found no significant association between maternal age or tobacco use and the risk of PTB [6,30,31].

This study has certain limitations. First, it was designed as a single-center retrospective study. Only cases with complete clinical and ultrasound data and confirmed delivery outcomes were included, which may have introduced selection bias. Data collection based on electronic medical records, which may have included incomplete or inaccurate information. Second, the relatively small sample size limited our ability to perform multivariate analyses to control for potential confounders such as indication, gestational age at cerclage placement, and progesterone use, which may have influenced the outcomes. In addition, the interval between cerclage and post-cerclage ultrasound was not standardized, which may have affected measurement consistency given the dynamic nature of cervical changes in high-risk pregnancies. Moreover, this study included three cerclage indications (history-based, ultrasound-indicated, and exam-indicated). The exam-indicated group had a higher rate of spontaneous PTB, which may have influenced the overall results. Additionally, transvaginal cervical length measurements are subject to inter-operator and inter-observer variability. Despite growing evidence that male fetuses are more susceptible to adverse intrauterine environments and have a higher risk of preterm birth, subgroup analyses by fetal sex were not performed due to the limited sample size [32]. Nonetheless, a major strength of our study is that all cervical length measurements were obtained transvaginally, which provides higher reliability and is less influenced by fetal position or maternal factors such as obesity. Furthermore, a single surgical technique (the McDonald method) was uniformly employed for all cerclage procedures, which enhances procedural consistency and minimizes technical variability across the study population.

## 5. Conclusions

Our study contributes to the existing body of evidence by demonstrating that specific findings on post-cerclage transvaginal ultrasound—particularly short cervical length, reduced residual cervical length above the cerclage, the presence of cervical funneling, and intra-amniotic sludge—are significantly associated with an increased risk of PTB before 34 weeks of gestation. Despite inherent limitations such as its retrospective design and relatively small sample size, our results demonstrate the potential benefit of TVUS in predicting PTB following cerclage placement. Further prospective, multicenter studies are needed to validate our findings and to establish standardized ultrasound criteria for risk assessment following cerclage.

## Figures and Tables

**Table 1:** Maternal demographics, baseline obstetric variables, and cerclage characteristics compared between women who delivered < 34 weeks and those at ≥34 weeks.

Characteristics, *n* = 225	Births ≥ 34 weeks (*n* = 28)	Births < 34 Weeks (*n* = 17)	*p*
Age (years)	31.15 ± 6.52	31.80 ± 5.80	0.719
BMI (kg/m^2^)	30.46 ± 6.32	31.28 ± 7.19	0.704
Gravity	3.0 (2.0–4.0)	3.5 (2.0 ± 7.75)	0.477
Parity	1.5 (0–2.0)	1.0 (0 ± 3.5)	0.781
Miscarriage	0.5 (0–1.0)	0.5 (0–4.0)	0.371
Smoking (*n*, %)	4 (14%)	2 (11.8%)	0.890
History of PTB in multiparous (*n*, %)	19 (57.9%)	10 (50.8%)	0.539
GA of earliest prior birth (wk)	26 ± 6.6	25.6 ± 5.5	0.877
GA at cerclage placement (wk)	15.6 ± 3.4	16.1 ± 4.4	0.381
Indication for cerclage placement			0.591
History-indicated	25 (89.3%)	9 (52.9%)	
Ultrasound-indicated	1 (3.6%)	3 (17.6%)	
Physical exam-indicated	2 (7.1%)	5 (29.4%)	
Progesterone use	22 (78%)	15 (88.2%)	0.690
Progesterone			0.306
Vaginal	14 (63.6%)	7 (46%)	
Intramuscular	8 (36.4%)	8 (53%)	

Abbreviations: PTB, preterm birth; GA, gestational age; BMI, body mass index. Note: Data are n (%), median (interquartile range), or mean ± standard deviation. A *p*-value < 0.05 indicates statistical significance.

**Table 2:** Transvaginal sonographic parameters at the first follow-up after cerclage, compared between women who delivered < 34 weeks and those at ≥34 weeks.

	Births ≥ 34 Weeks *n* = 28	Births < 34 Weeks *n* = 17	*p*
GA at ultrasound measurement (wk)	18.6 ± 4.5	19.6 ± 4.2	0.629
Interval from cerclage to first ultrasound (day)	14.0 (7.0–35)	14.0 (3.0–28.0)	0.675
Cervical length (mm)	35.89 ± 7.09	27.60 ± 8.81	0.012
Cervical length above the stitch (mm)	21.87 ± 8.95	13.15 ± 9.17	0.004
Cervical length below the stitch (mm)	13.65 ± 6.21	13.84 ± 6.28	0.921
Uterocervical angle between AUC-ECO (degrees)	103.71 ± 19.48	104.36 ± 18.27	0.920
Uterocervical angle between AUC-ICO (degrees)	105.16 ± 32.32	118.64 ± 26.87	0.194
Anterior cervical width at level of stitch (mm)	10.20 ± 3.83	9.76 ± 3.54	0.797
Posterior cervical width at level of stitch (mm)	11.57 ± 3.31	10.89 ± 3.19	0.668
Anterior stitch depth (mm)	7.61 ± 2.47	6.12 ± 1.12	0.184
Posterior stitch depth (mm)	6.64 ± 2.34	5.25 ± 2.70	0.249
Funneling membranes to or past level of stitch	0 (0%)	5 (%29.4)	0.005
Funneling membranes at level of internal os	1 (3.6%)	10 (58.8%)	<0.001
Intra-amniotic sludge present	1 (3.6%)	5 (29.4%)	0.023

Abbreviations: GA, gestational age; AUC-ECO, anterior uterine wall and external cervical os; AUC-ICO, anterior uterine wall and internal cervical os. Note: Data are *n* (%) or mean ± standard deviation. A *p*-value < 0.05 indicates statistical significance.

## Data Availability

The data that support the findings of this study are available on request from the corresponding author. The data are not publicly available due to privacy or ethical restrictions.
